# Effects of energy-protein supplementation frequency on performance of primiparous grazing beef cows during pre and postpartum

**DOI:** 10.5713/ajas.19.0784

**Published:** 2020-02-25

**Authors:** Felipe Henrique de Moura, Thaís Correia Costa, Aline Souza Trece, Luciano Prímola de Melo, Marcos Rocha Manso, Mário Fonseca Paulino, Luciana Navajas Rennó, Mozart Alves Fonseca, Edenio Detmann, Mateus Pies Gionbelli, Marcio de Souza Duarte

**Affiliations:** 1 Department of Animal Sciences, Universidade Federal de Viçosa, Viçosa, MG 36570-000, Brazil; 2 Muscle Biology and Nutrigenomics Laboratory, Universidade Federal de Viçosa, Viçosa, MG 36570-000, Brazil; 3 Department of Agriculture, Nutrition and Veterinary Sciences, College of Agriculture, Biotechnology & Natural Resources, University of Nevada – Reno, Reno, NV 89557, USA; 4 Department of Animal Science, Universidade Federal de Lavras, Lavras, MG 37200-000, Brazil

**Keywords:** Intake, Metabolism, Nitrogen Recycling, Periparturient Period, Tropical Pastures

## Abstract

**Objective:**

Twenty-four pregnant Nellore primiparous grazing cows were used to evaluate the effects of energy-protein supplementation and supplementation frequency during pre (105 d before calving) and postpartum (105 d after calving) on performance and metabolic characteristics.

**Methods:**

Experimental treatments consisted of a control (no supplementation), daily supplementation (1.5 kg/d of concentrate/animal) and infrequent supplementation (4.5 kg of concentrate/animal every three days). During the pre and postpartum periods, concentrations of blood metabolites and animal performance were evaluated. Ureagenesis and energy metabolism markers were evaluated at prepartum period.

**Results:**

Supplementation frequency did not alter (p>0.10) body weight (BW), average daily gain (ADG), and carcass traits during pre and postpartum. The BW (p = 0.079), adjusted BW at day of parturition (p = 0.078), and ADG (p = 0.074) were greater for supplemented cows during the prepartum. The body condition score (BCS; p = 0.251), and carcass traits (p>0.10) were not affected by supplementation during prepartum. On postpartum, supplementation did not affect animal performance and carcass traits (p>0.10). The dry mater intake was not affected (p>0.10) by supplementation and supplementation frequency throughout the experimental period. Daily supplemented animals had greater (p<0.001) glucose levels than animals supplemented every three days. Supplementation and supplementation frequency did not alter (p>0.10) the levels of blood metabolites, neither the abundance of ureagenesis nor energy metabolism markers.

**Conclusion:**

In summary, our data show that the reduction of supplementation frequency does not cause negative impacts on performance and metabolic characteristics of primiparous grazing cows during the prepartum.

## INTRODUCTION

The most important factors that limit the production efficiency of a cow-calf enterprise are reproduction and nutrition [1]; thus, due to additional demands for growth, together with the stress of first gestation and lactation, primiparous cows have contributed to production inefficiency [2]. Energy-protein supplementation is often required to improve the performance of grazing primiparous beef cows.

Supplementation programs substantially increase production costs in beef cattle systems, including expenses associated with the purchase of feed and the labour required for daily supplemental feeding [3]. Feeding strategies that reduce labor for supplemental feeding, such as providing supplement every three days, can be attractive to cow-calf producers due to the reduction in feeding and labor costs. The nitrogen recycling capacity of ruminants has been reported as the main biological mechanism that allows the use of infrequent supplementation [4]. This can be significant even when animals are fed medium-high quality forage [5]. Moreover, because the last trimester of gestation usually coincides with the dry season and, postpartum interval plays an important role in determining a primiparous cow’s calving interval in tropical conditions, producers are usually oriented to supplement cows at first and last trimester of gestation [6].

We hypothesized that the frequency of energy-protein supplementation can be reduced without impacting the performance and metabolic characteristics of beef primiparous cows. The aim of the current study was to evaluate the effects of energy-protein supplementation and supplementation frequency during prepartum (105 d from parturition) and postpartum (105 d after parturition) periods on the performance and metabolic characteristics of grazing primiparous beef cows.

## MATERIALS AND METHODS

### Animal care

The experimental procedure was approved by the Institutional Animal Care and Use Committee at Universidade Federal de Viçosa (19/2017).

### Experimental area

The experiment was carried out at the Department of Animal Science of the Universidade Federal de Viçosa, MG, Brazil, over the dry season, the dry-to-rainy transition season, and the rainy season. The climate of the area was classified according to Köppen-Geiger [7] as Cwa (humid temperate, with dry winter, hot summer).

### Animal management and experimental treatments

Twenty-four pregnant Nellore primiparous cows with an average body weight (BW) of 409±8.0 kg, 22±0.9 mo old, and at 172±2.5 d of gestation were used. The animals were managed on an experimental area of *Brachiaria decumbens* pastures divided into six paddocks of 4-ha for grazing with continuous stocking. Cows had unlimited access to water and mineral mix (87 g/kg calcium, 90 g/kg phosphor, 187 g/kg sodium, 90 g/kg sulphur, 2,400 mg/kg zinc, 800 mg/kg copper, 1,600 mg/kg manganese, 40 mg/kg iodine, 8 mg/kg cobalt, and 8.16 mg/kg selenium).

The experimental treatments were control (no concentrate supplement was fed); daily supplementation (1.5 kg/d/cow of supplement); and infrequent supplementation (4.5 kg/cow of supplement every three days; formulation of the concentrate supplement was outlined to avoid any potential acidosis [8]. The energy-protein supplement on an as-fed basis was composed of 425 g/kg wheat meal, 213 g/kg corn meal, 332 g/kg soybean meal, 27 g/kg urea, and 3 g/kg ammonium sulphate. The chemical composition of the experimental supplement is shown in Table 1.

A quantity of supplement (1.5 kg/d/cow) containing 450 g of crude protein (CP) was chosen to meet the protein requirements of primiparous cows, with an average gain of 0.6 kg/d at pasture with approximately 60 g/kg CP (dry matter [DM] basis) in order to produce mature weight female zebu cattle according to recommendations of Nutrient Requirements of Zebu and Crossbred Cattle (BR-CORTE) [9] upon parturition.

### Animal data collection

The animals were weighed at the beginning of the trial (105 d before calving), 15 d before parturition, at calving, and at the end of the trial (105 d after calving). Calf BW was recorded at birth. The BWs were obtained at were at 0600 h, except on the day of parturition. Calf BW was also recorded at birth and at 105 d after parturition. Upon analysis the BW were corrected to shrunk BW [10] in order to avoid possible confounding effect of digest:

$$\text{SBW}=0.8084\times {\text{BW}}^{1.0303}$$

Where, SBW is the shrunk body weight (kg) and BW is the body weight (kg).

Ribeye area (RA), fat thickness over the longissimus dorsi (between the 12th and the 13th ribs) and fat thickness over the biceps femoris muscle were recorded with an ultrasound (Aloka SSD 500; 3.5 MHz linear probe; Aloka Co. Ltd., Wallingford, CT, USA). Images were analysed in the BioSoft Toolbox II for Beef software (Biotronics Inc., Ames, Iowa, USA). In the morning of the same day that the ultrasound was performed, the body condition score (BCS) was recorded by three experienced technicians on a scale ranging from 1 to 9.

### Feed sampling and chemical analysis

Representative samples of supplement were collected monthly. Pasture sample compositions were obtained by hand-clipping every two weeks. Once a month, a second pasture sample was collected from each paddock. The second samples consisted of four forage subsamples randomly selected using a metal (0.5×0.5 m) square; the pasture was clipped approximately 1 cm above the ground to estimate the potentially digestible forage dry matter (pdDM) availability according to Paulino et al [11]. Samples of supplement and pasture were oven-dried (55°C) and ground in a Wiley mill (model 3; Arthur H. Thomas, Philadelphia, PA, USA) to pass through a 2-mm screen. Half of each ground sample was ground again to pass through a 1-mm screen.

The pdDM was estimated using the second pasture sam ple collected in each period as previously described, using the following equation [11]:

pdDM (%,dry matter basis)=0.98×(100-NDF)+(NDF-iNDF)

where 0.98 represents the true digestibility coefficient of the forage cell content; NDF represents the neutral detergent fiber assayed with a heat stable amylase; and iNDF is the forage content of indigestible neutral detergent fiber.

Two 9-d intake-digestibility trials were performed through out the experimental period, the first at 55 d before parturition and the second at 55 d after parturition. Titanium dioxide (TiO_2_) was used as an external marker to estimate fecal excretion (FE) [12]. Twenty grams of TiO_2_ per animal was packaged in paper cartridges and delivered via the oesophagus with a metal probe, once daily at 1030 h over nine days. Six days were allowed for stabilization of the external marker excretion, and fecal samples were collected at 0800 h and 1500 h on the seventh day, at 1000 h and 1700 h on the eighth day, and at 0600 h and 1300 h on the ninth day of the intake trial. Approximately 300 g of fecal sample was collected immediately after spontaneous defecation. Each fecal sample was oven-dried (55°C) and ground as described for pasture. Ground samples were proportionally mixed to make a single representative sample per animal on the pre- and postpartum.

Pooled samples of each material ground through 1-mm screen (supplement, pasture, and feces) were analysed according to the standard analytical procedures of the Brazilian National Institute of Science and Technology in Animal Science (INCT-CA; [13]; for DM (dried overnight at 105°C; method INCT-CA number G-003/1), ash (complete combustion in a muffle furnace at 600°C for 4 h; method INCT-CA number M-001/1), N (Kjeldahl procedure; method INCT-CA number N-001/1), ether extract (method INCT-CA number G-004/1), and NDF corrected for ash and protein (apNDF, using a heat-stable α-amylase, omitting sodium sulphite and correcting for residual ash and protein; method INCT-CA number F-002/1). The fecal samples were also analysed for levels of TiO_2_ by colorimetric (method INCT-CA M-007/1). From samples of supplement, pasture, and feces processed through a 2-mm screen, the iNDF content was determined as the residual NDF remaining after 288 h of ruminal *in situ* incubation using F57 filter bags (Ankom Technology Corp., Macedon, NY, USA), according to Valente et al [14].

The FE was estimated by the ratio of TiO _2_ and its concentration in the feces. The DM intake was estimated by using the iNDF as an internal marker and calculated by the following equation:

$$\text{DM }(\text{kg}/\text{d})=([(\text{FE}\times {\text{iNDF}}_{\text{feces}})-{\text{iNDF}}_{\text{sup}}]/{\text{iNDF}}_{\text{forage}})+\text{DMSI}$$

where FE is the fecal excretion (kg/d); iNDF_feces_ is the concentration of iNDF in the feces (kg/kg); iNDF_sup_ is the iNDF in the supplement (kg); iNDF_forage_ is the concentration of iNDF in forage (kg/kg); and DMSI is the DM supplement intake (kg).

### Blood hormone and metabolite assessment

Blood samples were collected in the peripartum period, critical period of physiological changes, by puncture of the jugular vein only at one single day, after 3 days of the infrequent supplementation and before the next infrequent supplementation at 0700 h at 27 d and 9 d prior to parturition, at the calving day, 9 d and at 27 d after parturition. Blood was collected into vacutainers with gel for serum separation and clot activation (BD Vacuntainer SST II Plus, São Paulo, Brazil) for analyses of insulin-like growth factor-1 (IGF-1), non-esterified fatty acids (NEFA), β-hydroxybutyrate (β-OHB), cholesterol, triglycerides, total protein, albumin, and urea. A second blood sample was collected in a second tube with ethylenediamine tetraacetic acid (EDTA) and sodium fluoride (BD Vacutainer Fluoreto/EDTA, São Paulo, Brazil) for glucose analysis. Both tubes were centrifuged at 2,700×*g* for 20 min. Following centrifugation, the plasma and serum were collected and subsequently frozen at −20°C for further analysis. Immediately after the centrifugation, a sample of plasma was collected to assess the concentration of free amino acids (AA).

Serum concentrations of IGF-1 were analysed by chemi luminescence using a Liaison analyser and Diasorin kit (DiaSorin, Saluggia, Italy). The levels of NEFA were quantified by the colorimetric method, and β-OHB was analysed by the kinetic enzymatic method based on the oxidation of D-3-hydroxybutyrate to acetoacetate (Ref. Numbers FA115 and RB1007 respectively, Randox, Ireland, UK). The concentration of free AA in serum was obtained using the high-performance liquid chromatography techniques described by Pitta et al [15]. Glucose (K082, Bioclin Quibasa, Belo Horizonte, Brazil), cholesterol (K083, Bioclin Quibasa, Brazil), triglycerides (K117, Bioclin Quibasa, Brazil), and urea (K056, Bioclin Quibasa, Brazil) were quantified by the enzymatic-colorimetric method and total protein (K031, Bioclin Quibasa, Brazil); albumin (K040, Bioclin Quibasa, Brazil) was analysed by the colorimetric method. All the analyses previously mentioned were determined by an automated biochemical analyser (Mindray BS 200E, Shenzhen, China). Globulins were calculated by subtracting the albumin quantified from the total protein level.

### Hepatic tissue and skeletal muscle biopsy

Biopsies of hepatic and skeletal muscle tissue were performed on the 27th day prior to calving. Six animals from each treatment were randomly selected for biopsies.

Liver sampling was performed via needle biopsy (Tru-Cut biopsy needle; Care Fusion Corporation, San Diego, CA, USA) 4 h before supplement feeding according to the procedure described by Mølgaard et al [16]. The incision was made between the 11th and 12th ribs from the right hepatic lobe [17]. Skeletal muscle sampling was performed on the left side at the 13th rib, three-fifths of the distance from the medial to the lateral edge of the longissimus muscle. Immediately, the liver samples (100 mg of tissue) and skeletal muscle samples (1 cm^3^) were placed in cryotubes, frozen and stored in liquid nitrogen at −196°C until processing.

### Abundance of carbamoyl phosphate synthase and mRNA expression of skeletal muscle energy metabolism markers

Whole liver protein was extracted in lysis buffer (10 mM Tris, pH 7.2; 0.5% Triton X-100; 10% glycerol; 0.5% dithiothreitol; 0.5 mM phenylmethanesulfonyl fluoride and 0.5 mM benzamidine). The protein content was measured with the Bradford Protein Assay (Bio-Rad, Hercules, CA, USA), and an equal amount of protein was separated with a 10% dodecyl sulphate-polyacrylamide gel electrophoresis. Proteins were transferred to nitrocellulose membranes and treated with blocking solution (3% bovine serum albumin w/v in tris-buffered saline with triton-X100 solution - TBSt) for 1 h with gentle agitation at room temperature. Membranes were then incubated with the following primary antibodies against carbamoyl phosphate synthase-1 (CPS-1 no. SC-376190, Santa Cruz, Dallas, TX, USA). The primary antibody was incubated at a 1:500 dilution in the blocking solution for 16 h at 4°C with gentle agitation. After incubation with the primary antibody, the membranes were washed 3 times at room temperature with TBSt and then incubated with the appropriate horseradish peroxidase secondary antibody (goat anti-mouse) at 1:5,000 dilution, for 1 h at room temperature with gentle agitation. Then, the membranes were washed 3 times (5 min each) with TBSt, developed with Clarity TM ECL substrate (Bio-Rad, USA), scanned with a c-Digit Blot scanner, and analysed with Image Studio (LI-COR Inc., Lincoln, NE, USA). The band density of target proteins was normalized using the density of bands of the load control samples that were handled and loaded under the same conditions as the target samples.

Total RNA (1 μg) was extracted from 0.5 g of muscle tissue samples using Trizol reagent (Invitrogen, Carlsbad, CA, USA). The RNA integrity (RIN) was evaluated by capillary electrophoresis using an RNA 6000 Nano kit and a 2100 Bioanalyser System (Agilent Techonologies, Santa Clara, CA, USA). Samples with RIN >7.0 were treated with DNAse I, Amplification Grade (Invitrogen, USA) and reverse transcribed into cDNA using the GoScript Reverse Transcription System (Promega, Madison, WI, USA). The *mRNA* levels of carnitine palmitoyl transferase 1 (*CPT-1*) were quantified using the following primers: Forward - GTCCCTTCCCTTGCTCTA, Reverse - GGACAGCAGAGACCCATA, while the *mRNA* expression of peroxisome proliferator-activated receptor γ coactivator 1 α (*PGC-1α*) was quantified using the following primers: Forward -GAAGCGGGAATCCGAAAG, Reverse - CTCAGTT CTGTCCGTGTTG. The housekeeping gene used was 18S, which was quantified using the following primer: Forward - CCTGCGGCTTAATTTGACTC, Reverse - AACTAAGAA CGGCCATGCAC. A quantitative polymerase chain reaction (qPCR) was performed on a 7300 Real-Time PCR System (Applied Biosystems, Carlsbad, CA, USA) using a GoTaq kit (Promega, USA) and the following cycle parameters: 95°C for 3 min and 40 cycles at 95°C for 10 s and 60°C for 30 s. The amplification efficiency ranged from 0.90 to 0.99. After amplification, a melting curve (0.01°C/s) was used to confirm product purity. Relative gene expression data was calculated as described by Livak and Schmittgen [18].

### Statistical analysis

Statistical analyses were performed using PROC MIXED in SAS 9.4 (SAS Inst., Cary, NC, USA) and analysed according to a completely randomized design. The groups of animals were considered the experimental units, as per the following statistical model:

$${\text{Y}}_{\text{ijk}}=\mu +{\text{T}}_{\text{i}}+{\text{G}}_{(\text{i})\text{j}}+\in_{(\text{ij})\text{k}}$$

where Y_ijk_ is the observation taken on subject k in experimental unit j undergoing treatment I; μ is the overall constant; T_i_ is the effect of treatment i (fixed effect); G_(i)j_ is the effect of the group nested to the treatment i (random effect); and ∈_(ij)k_ is the unobservable random error associated with each observation.

Contrasts were constructed in order to evaluate the effects of supplementation (contrast between cows supplemented daily + cows infrequently supplemented vs non-supplemented cows) and frequency of supplementation (contrast between cows supplemented daily versus cows supplemented every three days). Due to the high probability of type II error, α = 0.10 was adopted. Initial BW and BCS were used as covariates. The choice of the best (co)variance matrix was performed following the Akaike information criteria with correction. The degrees of freedom were estimated according to the Kenward-Roger method. The blood IGF-1 and metabolites variables were evaluated as repeated measures over time [19].

## RESULTS

### Intake, digestibility, and animal performance

Available forage and forage chemical composition in the pre- and postpartum periods are shown in Table 1. The animals grazed medium quality forage during pre-and postpartum (CP >70 g/kg of DM and apNDF <60 g/kg of DM) [8].

Supplementation frequency did not alter (p >0.10) DM intake (kg/d and g/kg BW), forage dry matter intake (FDM; kg/d and g/kg BW), organic matter (OM; kg/d and g/kg BW), digested OM (kg/d), apNDF (kg/d and g/kg BW), digested NDF (kg/d and g/kg BW), iNDF (kg/d and g/kg BW), CP (kg/d), CP (g/kg of BW) and CP:dOM (g/kg – Table 2; p< 0.10) in the pre- and postpartum periods. In the prepartum period, there was a greater intake of CP (kg/d and g/kg of BW; p<0.01), digested OM (dOM; p = 0.065), and CP:dOM ratio (p = 0.002) for supplemented cows than for cows in the control treatment. However, the supplementation did not alter DM intake (p = 0.288), FDM intake (p = 0.245), OM (p = 0.255), apNDF (p = 0.756), iNDF (p = 0.311) and digested NDF (dNDF; p = 0.971).

In the postpartum period, a greater intake of CP (kg/d, p = 0.02; and g/kg of BW, p<0.01) and CP:dOM ratio (p = 0.039) were observed for supplemented cows compared to cows from the control treatment. No differences were observed between supplemented and non-supplemented cows for DM intake (p = 0.484), FDM intake (p = 0.487), OM (p = 0.454), apNDF (p = 0.685), iNDF (p = 0.871), dOM (p = 0.374) and dNDF (p = 0.587).

A greater CP digestibility (p = 0.002), OM digestibility (p = 0.057) and dietary concentration of dOM (p = 0.043) were observed for supplemented cows compared to cows from the control treatment. No difference was observed between supplemented and non-supplemented cows for apNDF digestibility (p = 0.650). Likewise, the supplementation frequency did not alter (p>0.10) the digestibility (Table 3) of OM (g/g), CP (g/g), apNDF (g/g), and dietary concentration of dOM (g/kg) in the pre- and postpartum periods. There was greater CP digestibility (p = 0.006) for supplemented cows compared to cows from the control treatment. On the other hand, no differences were observed between supplemented and non-supplemented cows for OM digestibility (p = 0.241), apNDF digestibility (p = 0.546) and dietary concentration of dOM (p = 0.204).

The supplementation frequency did not alter (p >0.10; Table 4) BW, adjusted BW at day of parturition (adjBW), BW after calving upon parturition (calvingBW) average daily gain (ADG), BCS, RA, fat-thickness on the longissimus muscle (FAT-Ld) and on the biceps femoris muscle (FAT-Bf) during the pre- and postpartum periods.

There was a supplementation effect at 15 d before calving for BW (p = 0.079) and ADG (p = 0.074). The RA (p = 0.352), FAT-Ld (p = 0.199) and FAT-Bf (p = 0.924) were not affected by supplementation 15 d before calving.

The adjBW (p = 0.078) was higher for supplemented cows at parturition day. The calving BW and BCS at parturition day were not affected (p>0.10) by supplementation and supplementation frequency.

The supplementation did not affect (p >0.10) BW, ADG, BCS, RA, FAT-Ld, and FAT-Bf at 105 d postpartum. Furthermore, the birth BW of calves was not different (p>0.10; Table 4) according to supplementation and supplementation frequency.

### Hormone and metabolite levels

The IGF-1 level (p = 0.744; Table 5) and glucose concentration (p = 0.865; Table 5) were similar between supplemented and non-supplemented cows. The reduction of supplementation frequency did not alter (p = 0.368) IGF-1 levels. However, the glucose concentration was greater (p<0.001) for cows supplemented daily than that in cows supplemented infrequently, and there was a sampling time effect for glucose concentration (p<0.001; Table 5; Supplementary Figure S1).

**Figure 1:**
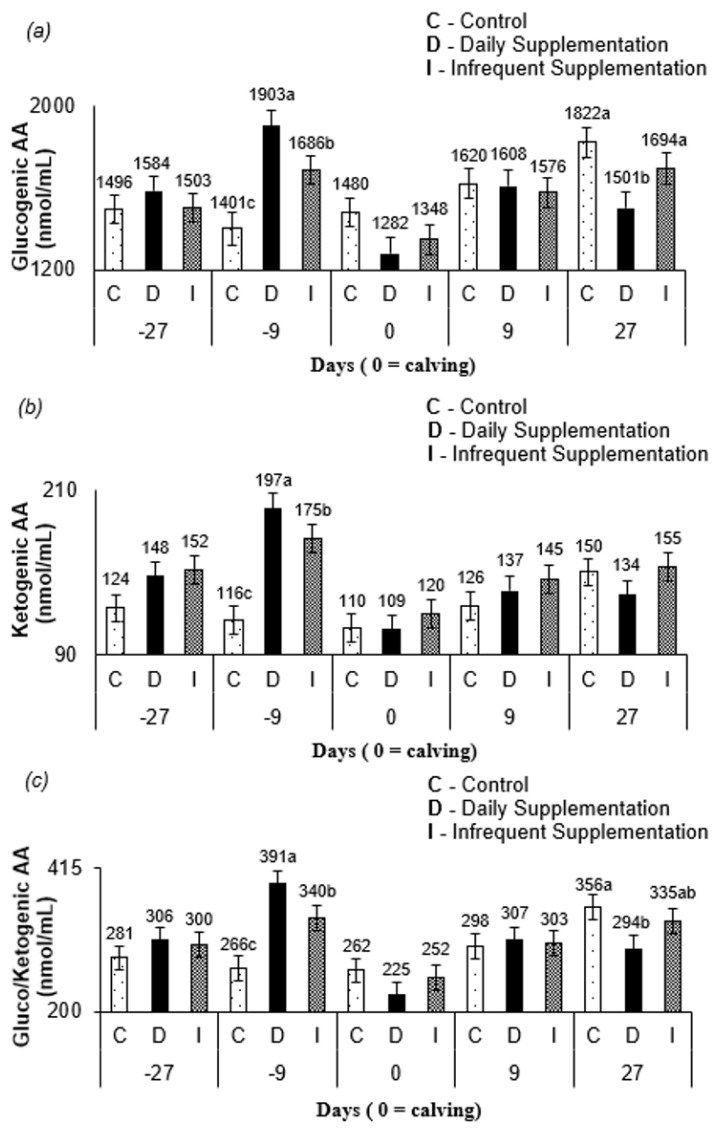
Serum concentration of (a) free amino acids and (b) serum urea nitrogen as a function of the days of sampling and the different treatments. Blood samples were collected 27 d and 9 d prior to parturition, at the calving day (day 0), 9 d, and 27 d after parturition from cows receiving the treatments control (C), daily (D) and infrequent (I) supplementation. Free amino acids and urea concentrations were obtained from blood serum and serum urea nitrogen (SUN) was estimated as 46.7% of total urea. Least square means within the sampling days followed by different letters are different (p<0.10). There were significant differences in free amino acids concentrations between treatments at 9 d prior and 27 d after parturition. While, significant differences were observed regarding the serum urea nitrogen at 27 d and 9 d prior to parturition and in the calving day.

There was an interaction between treatment and sampling time for free AA (p<0.001; Table 5) and serum urea nitrogen (SUN; p = 0.005; Table 5). At 9 d before calving, daily supplemented cows had higher (p<0.10; Figure 1a) free AA than cows from infrequent supplementation treatment, and there were greater concentrations of free AA for animals infrequently supplemented than that in non-supplemented cows. At 27 d postpartum, free AA levels were greater (p<0.10) for cows from control and infrequent treatment compared to daily supplemented cows. Supplemented cows had greater (p<0.10; Figure 1b) SUN levels than non-supplemented cows at 27 d and 9 d before calving and at parturition.

**Figure 2:**
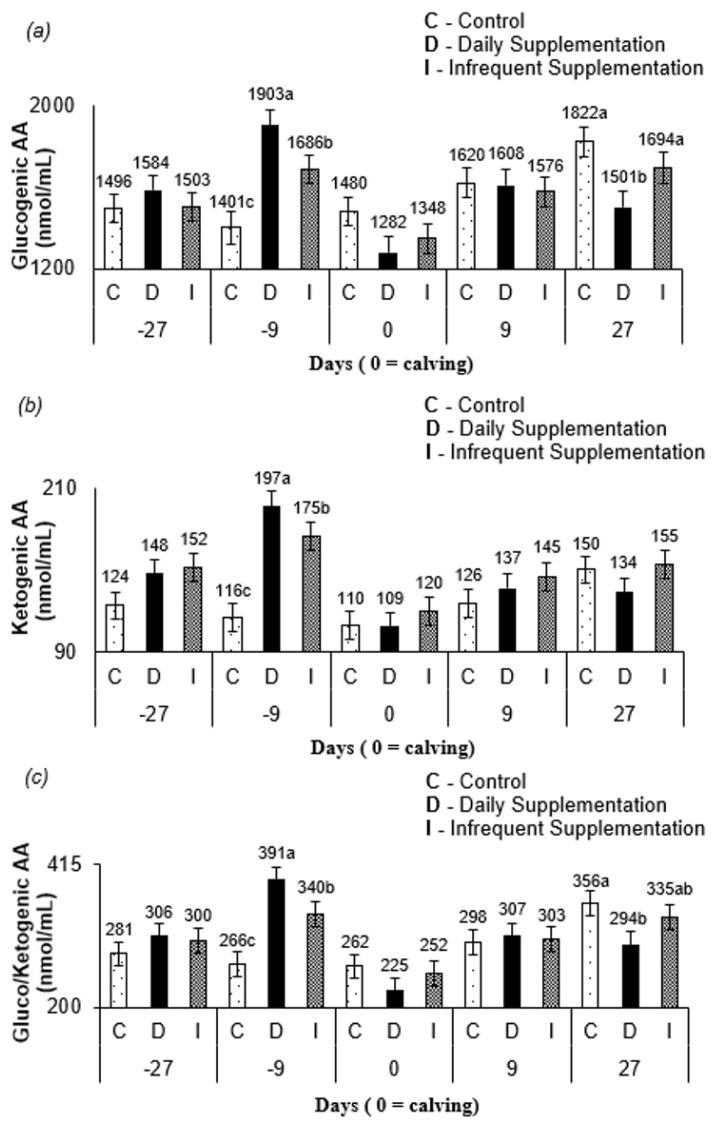
Serum concentration of (a) glucogenic amino acids, (b) ketogenic amino acids and (c) gluco/ketogenic amino acids as a function of the days of sampling and the different treatments. Blood samples were collected 27 d and 9 d prior to parturition, at the calving day (day 0), 9 d, and 27 d after parturition from cows receiving the treatments control (C), daily (D) and infrequent (I) supplementation. Least square means within the sampling days followed by different letters are different (p<0.10). There were significant differences in glucogenic and gluco/ketogenic amino acids concentrations between treatment at 9 d prior and 27 d after parturition. Ketogenic amino acids concentrations only differ between treatments at 9 d prior to parturition.

An interaction between treatment and sampling time was observed for glucogenic AA (p<0.001), ketogenic AA (p<0.001), and gluco/ketogenic AA (p<0.001). At 9 d before parturition, cows supplemented daily had higher (p< 0.10; Figure 2a, 2b, 2c) glucogenic AA, ketogenic AA, and gluco/ketogenic AA, and there were greater concentrations of glucogenic AA, ketogenic AA, gluco/ketogenic AA for animals infrequently supplemented than those in non-supplemented cows. At 27 d postpartum, glucogenic AA and gluco/ketogenic AA were greater (p<0.10) for cows from control and infrequent treatments compared to those in cows supplemented daily.

No effects of supplementation (p = 0.980) and supplemen tation frequency (p = 0.366) were observed on NEFA serum levels. Likewise, the supplementation (p = 0.402) and the reduction of supplementation frequency (p = 0.207) did not alter β-OHB serum levels. There was a sampling time effect (p<0.001) for NEFA and β-OHB serum levels (Supplementary Figure S2a, S2b).

Supplementation did not alter the serum concentrations of total protein (p = 0.122), albumin (p = 0.406) and globulins (p = 0.221). Furthermore, there was not an observed effect of supplementation frequency on total protein (p = 0.198), albumin (p = 0.795), and globulins (p = 0.678). There was a sampling time effect (p<0.001) on the serum concentration of total protein and globulins (Supplementary Figure S3a, S3b).

Cholesterol (p = 0.762) and triglycerides levels (p = 0.973) were similar between supplemented cows and non-supplemented. Likewise, no differences were observed between daily supplemented and infrequently supplemented cows for cholesterol (p = 0.869) and triglyceride concentrations (p = 0.398). There was a sampling time effect (p<0.001) on the serum levels of cholesterol and triglycerides (Supplementary Figure S4a, S4b).

### Abundance of carbamoyl phosphate synthase and mRNA expression of skeletal muscle energy metabolism markers

No effect of supplementation (p = 0.815; Table 6) and supplementation frequency (p = 0.987) was observed on the overall content of hepatic CPS-1 protein. Furthermore, the supplementation did not alter the mRNA abundance of *PGC-1α* (p = 0.433) and *CPT-1* (p = 0.273). Likewise, the reduction of supplementation frequency did not alter the mRNA abundance of *PGC-1α* (p = 0.365) and *CPT-1* (p = 0.164).

## DISCUSSION

The forage mass available was not a limiting factor of feed intake in this study (Table 1). The interpretation of forage available for grazing as a baseline nutritional resource should be conducted from the perspective of the fraction potentially convertible into animal product; this can be achieved through utilization of pdDM which integrates the quantity and quality regardless of season [11]. The overall average pdDM mass at prepartum (81 g/kg BW) and at postpartum period (89 g/kg BW) were higher than 40 to 50 g/kg BW for satisfactory intake and performance in a grazing system [20]; thus throughout the duration of the trial, the animals had the possibility of highly selective grazing and choosing the best-quality forage parts.

Although the provision of supplemental nitrogen has been reported to increase DM intake substantially [7], this pattern was not observed (Table 2). The adequacy of the dietary protein-to-energy ratio has been pointed out as one of the main indicators of the intake patterns of cattle fed tropical forages [21]. The maximum forage intake has been observed with dietary CP:dOM at approximately 216 g/kg [22]. Although there is a higher dietary CP:dOM for supplemented cows than non-supplemented cows during the pre- and postpartum periods (Table 2), the dietary CP:dOM observed in our study for supplemented cows was below the value suggested by Reis et al [22]. Thus, regardless of the treatment, all cows had low dietary protein-to-energy ratios (CP:dOM <216 g/kg) which seems to support an unaltered forage intake between supplemented and non-supplemented cows as well as between cows from different supplementation frequencies. However, similar FDM intake between cows supplemented daily and cows supplemented every three days is an indicator that the reduction of supplementation frequency can be attractive to cow-calf producers. Usually, infrequent supplementation under medium-high quality forage has been reported as a reduction in the forage voluntary intake [23].

Energy-protein supplementation increased the CP intake for supplemented cows due to the additional supply of protein provided by the supplement (Table 2). During the prepartum period, supplemented cows had a greater OM and CP digestibility compared to non-supplemented cows (Table 3), which resulted in a greater dOM intake for supplemented cows (Table 2). Such a pattern has been associated with the supplementation of the animals, since concentrates usually have a greater digestibility than forage [24]. However, during the postpartum period, the supplementation increased only the CP digestibility (Table 3). On the other hand, the reduction of supplementation frequency did not change intake (Table 2) and total apparent digestibility (Table 3). Such observations may be explained by the fact that the same quantity of supplement was offered to both treatments (daily and infrequent). Previous studies have demonstrated that cattle can efficiently recycle urea to supply nitrogen to the rumen [25, 26]. Thus, it is possible that animals from the infrequent group may have increased the urea recycling and kept the levels of nitrogen in the rumen at equivalent levels of daily supplemented animals, which may have contributed for a lack of change in forage intake between daily and infrequently supplemented animals.

The increase in dOM and CP intake increased the BW, ADG during the prepartum period (15 d before calving; Table 4), and adjBW at parturition (Table 4) for supplemented cows. Primiparous cows seem to be more sensitive to nutrient intake and consequently BCS changes more drastically than non-primiparous animals [8], but this pattern was not observed. In fact, regardless of treatment, all cows had a BCS between the minimum (5.0) and maximum (6.0) at calving which are acceptable values according to the NASEM [8] recommendation to allow the reproductive success of the animals during the breeding season. Our data suggest that non-supplemented cows may adapt their energy metabolism (e.g., maintenance energy) to periods of lower availability of nutrients [27] allowing them to maintain similar BCS, RA, and fat-thickness compared to supplemented cows on prepartum (Table 4). During the postpartum period, there was no significant dOM effect which ultimately produced similar BW, BCS, RA, FAT-Ld, FAT-Bf, and ADG between supplemented and non-supplemented cows (Table 4). Intake during the pre- and postpartum periods in cows supplemented daily and cows supplemented every three days was not significantly different (Table 2) and resulted in similar BW, adjBW, calvingBW, ADG, BCS, RA, FAT-Ld, FAT-Bf, and ADG (Table 4).

Maternal supplementation during the last trimester of pregnancy has been reported to be an important factor for fetal growth, altering calf birth weight [8]. However, in our study, no over- and underfeeding was observed among cows subjected to different strategies of supplementation (Table 2). Thus, similar calf BW for all treatments was observed (Table 4).

Nutrient intake pre- and postpartum influences concen trations of IGF-1 in serum of primiparous lactating beef cows [28]. Reduced nutrient intake uncouples the growth hormone (GH)–IGF-1 axis and it increases GH secretion in cattle; whereas serum concentrations of IGF-1 are decreased. The decline in circulating IGF-1 is paralleled by a decline in circulating insulin that stimulate hepatic gluconeogenesis providing glucose for the fetus or for lactose synthesis; additionally, NEFA is mobilized from adipose tissue to provide energy for peripheral needs [29]. Elevated NEFA levels can result in ketone body production, such as β-OHB, another energetic substrate [30]. Thus, the data presented by this current study demonstrate that the intake of nutrients through the peripartum period was enough to avoid problems concerning energy balance between supplemented and non-supplemented cows and between cows supplemented daily and cows supplemented every three days. The similar NEFA and β-OHB levels between non-supplemented and supplemented cows and between cows subjected to different supplementation strategies support such an argument (Table 5). Furthermore, NEFA levels during the peripartum period (Table 5; Supplementary Figure S2a) were below the threshold of 0.40 mmol/L, the value utilized by Lopes et al [31] as an indicative of problems with energy balance in grazing beef cows.

Cholesterol and triglyceride levels between supplemented and non-supplemented cows and between the studied frequency treatments were not different (Table 5). Thus, our data indicate that cows in all treatments were able to cope with the β-oxidation of NEFA and to export those not used as metabolic fuel.

The dietary intake of starch substrates is directly associated with greater hepatic gluconeogenesis in ruminants [32]. However, cows subjected to decreased supplementation frequency had low concentrations of glucose (Table 5). Since blood sampling occurred only on one single day, 3 days after infrequent supplementation and before the next supplementation, it is possible that plasma glucose concentrations decreased during the days that no supplementation was offered. Thus, no effects of supplementation on the plasma concentration of glucose between supplemented and non-supplemented cows were observed due the large variance in glucose levels between cows of different supplementation frequencies (Table 5).

Cows in a negative energy state increase body fat mobili zation followed by a high expression of the genes involved in fatty acid oxidation and utilization, such as *CPT-1* and *PGC-1α* [33,34]. Therefore, our data indicate a lack of effects on *CPT-1* and *PGC-1α mRNA* expression in skeletal muscle (Table 6) which suggests that the energy metabolism of skeletal muscle did not changed between treatments, which may explain the similar NEFA levels between supplemented and non-supplemented cows and between cows subjected to different supplementation frequencies.

A dependence on nitrogen recycling in the rumen is an indication of dietary protein deficiency [21]. This biological event can be significant even in medium-high quality forage and is evidenced by low SUN levels due to transfer of forage to the rumen to maintain microbial growth [5]. Furthermore, situations where nitrogen recycling occurs without myofibrillar protein mobilization [5,35], associated with a predominant use of absorbed AA for nitrogen recycling, may suggest a low peripheral circulation pool of free AA. Thus, at 27 and 9 d prepartum and at calving day, supplemented cows had higher SUN levels than non-supplemented cows (Figure 1b) due to the increase in dietary nitrogen intake associated with higher rates of ammonia transfer from the rumen, output of urea from the liver into the blood, and lower SUN transfer into the rumen [24]. However, the pool of free AA was higher only at 9 d before calving (Figure 1a) for supplemented cows. Thus, it is reasonable to believe that supplemented cows had enough escape of dietary protein to avoid the use of metabolizable AA for the synthesis of urea for recycling at 9 d before calving.

Lactation peaks in Nellore cows occur between the third and fourth postpartum week [9]. Thus, the higher blood AA at 27 d postpartum (Figure 1a) for cows from control and infrequent treatment compared to that in cows from daily treatment occurred to support milk production, since these treatments did not have a daily constant availability of energy and protein compounds via supplement. The greater amount of glucogenic AA (Figure 2a), a precursor of lactose synthesis for cows, from control and infrequent treatment at 27 d after parturition corroborates this conclusion. Furthermore, since the blood sampling occurred on the third day of the supplementation cycle, the AA mobilization likely increased through the days when no supplementation was offered for infrequently supplemented cows. The levels of glucogenic AA, ketogenic AA, and gluco/ketogenic AA (Figure 2c) for all treatments at 9 d prepartum and at 27 d postpartum may have occurred due to homeostatic AA concentration control [24]. The levels of the free AA pool pattern in all treatments (Figure 1a) are consistent with this argument.

Protein supplementation frequency and protein over- and underfeeding have previously been explored scientifically and reported with a greater action of enzymes involved in the urea cycle, such as CPS-1 [36,37]. The CP intake (Table 2) of non-supplemented cows was above the minimum level required to meet their maintenance requirements [9]. Likewise, daily supplemented cows had CP intakes below the threshold maximum at which positive responses to supplemental protein have been observed (225 g CP/kg DM) [21]. Thus, this may suggest that the similar values for hepatic CPS-1 abundance (Table 6) observed between supplemented and non-supplemented cows occurred because dietary protein was not extreme enough to alter CPS-1 abundance. This can explain the similar CPS-1 abundance between cows from different supplementation frequencies, since for the infrequent supplementation, the CP intake was approximately 200 g CP/kg DM (Table 2).

In summary, our data indicate that the reduction of sup plementation frequency (4.5 kg/cow of supplement every three days) does not negatively affect the performance and metabolic characteristics of primiparous cows under grazing conditions. Moreover, the results suggest that energy-protein supplementation during the pre- and postpartum periods of primiparous beef cows under grazing conditions likely decreased forage intake leading to a similar nutrients intake among treatments and no changes on animal performance.

## Figures and Tables

**Table 1:** Potentially digestible forage mass production, and chemical composition of forage and a supplement during pre and postpartum of period

Item1)	Prepartum		Postpartum		Supplement
	
	*Brachiaria decumbens*2)	*Brachiaria decumbens* (Intake trial)3)	*Brachiaria decumbens*2)	*Brachiaria decumbens* (Intake trial)3)	
pdDM (kg/ha)4)	2,560±33.0	2,346±205.0	2,518±186.0	2,807±362.0	-
OM (g/kg)	918±1.3	919±1.0	924±2.1	929±1.6	955.0
CP (g/kg)	69±2.2	73±1.7	73±2.8	68±3.1	300.0
NIDIN (g/kg of total N)	185±9.1	282±10.5	155±7.9	272±4.8	0.68
apNDF (g/kg)	586±14.1	546±9.3	564±6.9	568±12.9	186.0
iNDF (g/kg)	213±10.6	180±4.1	223±14.3	210±5.7	46.0

pdDM, potentially digestible forage dry matter; OM, organic matter; CP, crude protein; NIDIN, neutral detergent insoluble N; apNDF, neutral detergent fiber corrected for ash and protein residue; iNDF, indigestible neutral detergent fiber.

1) Chemical composition was evaluated in the hand-plucked forage sample.

2) Mean±standard error of the mean of pdDM and chemical composition of forage during the pre and postpartum period.

3) Mean±standard error of the mean of pdDM and chemical composition of forage during the pre and postpartum intake trial.

4) pdDM was estimated for forage sampled in the area delimited by a metal square 0.5×0.5 m.

**Table 2:** Intake according to frequency of supplementation during pre and postpartum of grazing primiparous beef cows

Items	Treatment			SEM	p-value1)	
	
	Control	Daily	Infrequent		C vs S	D vs I
Prepartum						
DM (kg/d)	7.69	8.46	8.17	0.39	0.29	0.64
FDM (kg/d)	7.69	7.13	6.84	0.39	0.24	0.64
OM (kg/d)	7.07	7.82	7.57	0.36	0.25	0.68
CP (kg/d)	0.52	0.98	0.98	0.03	<0.01	0.99
apNDF (kg/d)	4.19	4.14	4.03	0.25	0.76	0.78
iNDF (kg/d)	1.41	1.35	1.27	0.07	0.31	0.48
dOM (kg/d)	4.41	5.32	5.20	0.24	0.06	0.75
dNDF (kg/d)	2.69	2.72	2.64	0.23	0.97	0.82
CP:dOM (g/kg)	119	184	188	5.1	<0.01	0.61
DM (g/kg of BW)	17.9	19.7	19.2	0.95	0.27	0.72
FDM (g/kg of BW)	17.9	16.6	16.1	0.94	0.28	0.71
OM (g/kg of BW)	16.4	18.2	17.8	0.87	0.24	0.77
CP (g/kg of BW)	1.16	2.20	2.16	0.19	<0.01	0.74
apNDF (g/kg of BW)	9.7	9.7	9.5	0.60	0.83	0.83
iNDF (g/kg of BW)	3.3	3.1	3.0	0.17	0.36	0.54
Postpartum						
DM (kg/d)	8.49	9.15	9.17	0.68	0.48	0.98
FDM (kg/d)	8.49	7.82	7.84	0.68	0.49	0.98
OM (kg/d)	7.89	8.56	8.52	0.62	0.45	0.96
CP (kg/d)	0.58	0.95	1.03	0.07	0.02	0.49
apNDF (kg/d)	4.84	4.74	4.61	0.31	0.68	0.78
iNDF (kg/d)	1.73	1.81	1.63	0.07	0.87	0.21
dOM (kg/d)	4.20	4.67	4.98	0.49	0.37	0.69
dNDF (kg/d)	2.80	2.56	2.66	0.26	0.59	0.80
CP:dOM (g/kg)	137	205	211	16.5	0.04	0.81
DM (g/kg of BW)	19.9	21.0	21.8	1.64	0.52	0.78
FDM (g/kg of BW)	19.9	17.9	18.6	1.61	0.47	0.79
OM (g/kg of BW)	18.5	19.7	20.2	1.49	0.49	0.82
CP (g/kg of BW)	1.28	2.39	2.23	0.21	<0.01	0.33
apNDF (g/kg of BW)	11.4	10.9	11.0	0.82	0.67	0.95
iNDF (g/kg of BW)	4.1	4.1	3.9	0.17	0.68	0.35

SEM, standard error of the mean; DM, dry matter; FDM, forage dry matter; OM, organic matter; CP, crude protein; apNDF, neutral detergent fiber assayed with a heat stable amylase and corrected for residual ash and protein; iNDF, indigestible neutral detergent fiber; dOM, digested organic matter; dNDF, digested neutral detergent fiber; BW, body weight.

1) C vs S = contrast between cows supplemented daily + cows infrequently supplemented versus non supplemented cows; D vs I = contrast between cows supplemented daily versus cows supplemented every three days. Values differ significantly at p<0.10.

**Table 3:** Total apparent digestibility and dietary concentration of digested organic matter according to frequency of supplementation during pre and postpartum of grazing primiparous beef cows

Items	Treatment			SEM	p-value1)	
	
	Control	Daily	Infrequent		C vs S	D vs I
Prepartum						
OM (g/g)	0.63	0.68	0.69	0.16	0.06	0.89
CP (g/g)	0.45	0.69	0.70	0.02	<0.01	0.78
apNDF (g/g)	0.64	0.66	0.65	0.02	0.65	0.89
dOM (g/kg of DM)	575.0	632.0	638.0	14.3	0.04	0.79
Postpartum						
OM (g/g)	0.53	0.55	0.58	0.02	0.24	0.30
CP (g/g)	0.32	0.50	0.57	0.02	<0.01	0.15
apNDF (g/g)	0.58	0.54	0.57	0.02	0.55	0.40
dOM (g/kg of DM)	493.0	512.0	540.0	16.6	0.20	0.33

SEM, standard error of the mean; OM, organic matter; CP, crude protein; apNDF, neutral detergent fiber assayed with a heat stable amylase and corrected for residual ash and protein; dOM, digested organic matter; DM, dry matter.

1) C vs S = contrast between cows supplemented daily + cows infrequently supplemented versus non supplemented cows; D vs I = contrast between cows supplemented daily versus cows supplemented every three days. Values differ significantly at p<0.10.

**Table 4:** Performance according to frequency of supplementation during pre and postpartum of grazing primiparous beef cows

Items	Treatment			SEM	p-value1)	
	
	Control	Daily	Infrequent		C vs S	D vs I
Prepartum						
105 d before calving						
BW (kg)	411.0	408.0	409.0	14.5	0.91	0.98
BCS (1–9)	5.5	5.4	5.5	0.23	0.93	0.83
RA (cm^2^)	48.9	51.7	49.1	2.36	0.65	0.50
FAT-*Ld* (mm)	2.5	3.6	3.8	0.72	0.27	0.81
FAT-*Bf* (mm)	4.2	6.0	6.1	1.16	0.28	0.92
15 d before calving						
BW (kg)	436.0	451.0	443.0	3.3	0.08	0.18
ADG2) (kg/d)	0.35	0.54	0.43	0.04	0.07	0.18
RA (cm^2^)	51.0	49.2	51.0	0.60	0.35	0.13
FAT-*Ld* (mm)	3.2	3.8	3.5	0.17	0.19	0.36
FAT-*Bf* (mm)	5.7	5.9	5.4	0.16	0.92	0.15
Parturition						
adjBW3) (kg)	451.0	475.0	460.0	5.0	0.08	0.12
calvingBW (kg)	403.0	423.0	411.0	6.9	0.20	0.30
BCS (1–9)	5.7	6.0	6.0	0.20	0.25	0.95
calfBW (kg)	30.0	31.0	30.0	1.6	0.92	0.74
Postpartum						
105 d after calving						
BW (kg)	436.0	441.0	442.0	12.0	0.76	0.97
ADG4) (kg/d)	0.32	0.15	0.31	0.08	0.43	0.27
BCS (1–9)	5.3	5.5	5.6	0.19	0.30	0.76
RA (cm^2^)	51.5	49.6	52.7	1.67	0.89	0.30
FAT-*Ld* (mm)	3.2	4.0	3.9	0.57	0.45	0.94
FAT-*Bf* (mm)	5.6	6.0	4.8	0.59	0.81	0.23

SEM, standard error of the mean; BW, body weight; BCS, body condition score; RA, ribeye area; FAT-*Ld*, backfat-thickness on longissimus muscle; FAT-*Bf*, backfat-thickness on Biceps femoris muscle; ADG average daily gain; adjBW adjusted BW for day of parturition; calvingBW, BW after calving upon parturition; calfBW, calf body weight.

1) C vs S = contrast between cows supplemented daily + cows infrequently supplemented versus non supplemented cows; D vs I = contrast between cows supplemented daily versus cows supplemented every three days. Values differ significantly at p<0.10.

2) ADGprepartum = [(BW15 d before calving − BW105 d before calving) / 90 days].

3) adjBW = [BW15 d before calving + (ADGprepartum × number of days until parturition)].

4) ADGpostpartum = [(calvingBW − BW105 d after calving) / 105 days].

**Table 5:** Endocrine and metabolic profile measured in blood serum of grazing primiparous beef cows according to frequency of supplementation during pre and postpartum

Items	Treatment			SEM	p-value1)			
	
	Control	Daily	Infrequent		C vs S	D vs I	T	T×treat
Insulin-like growth factor-1 (ng/mL)	206.0	222.0	202.0	16.4	0.74	0.37	0.312	0.41
Glucose (mg/dL)	69.2	72.3	65.6	0.97	0.86	<0.01	<0.01	0.33
Free amino acids (nmol/mL)	1,689.0	1,717.0	1,712.0	51.4	0.66	0.94	<0.01	<0.01
Glucogenic amino acids (nmol/mL)2)	1,563.0	1,576.0	1,563.0	46.1	0.90	0.83	<0.01	<0.01
Ketogenic amino acids (nmol/mL)3)	127.0	145.0	150.0	7.7	0.11	0.67	<0.01	<0.01
Gluco/ketogenic amino acids (nmol/mL)4)	292.0	305.0	306.0	12.8	0.41	0.93	<0.01	<0.01
SUN5) (mg/dL)	10.6	17.3	13.9	1.11	0.03	0.11	<0.01	<0.01
Non-esterified fatty acids (mmol/L)	0.21	0.23	0.18	0.03	0.98	0.37	<0.01	0.62
β-hydroxybutyrate (mmol/L)	0.45	0.52	0.45	0.03	0.40	0.21	<0.01	0.64
Total protein (g/dL)	6.58	6.74	6.64	0.13	0.12	0.20	<0.01	0.81
Albumin (g/dL)	3.38	3.31	3.28	0.08	0.41	0.79	0.23	0.88
Globulins (g/dL)	3.19	3.43	3.36	0.17	0.22	0.68	<0.01	0.83
Cholesterol (mg/dL)	125.7	128.2	130.5	10.2	0.76	0.87	<0.01	0.96
Triglycerides (mg/dL)	26.6	27.8	25.2	1.92	0.97	0.40	<0.01	0.19

SEM, standard error of the mean.

1) C vs S = contrast between cows supplemented daily + cows infrequently supplemented versus non supplemented cows; D vs I = contrast between cows supplemented daily versus cows supplemented every three days; T = Time, days relative at calving; T×treat = interaction between sampling time and treatment. Values differ significantly at p<0.10.

2) Alanine; arginine; aspartate; asparagine; glutamate; glutamine; histidine; methionine; serine; valine.

3) Leucine.

4) Isoleucine; phenylalanine; tryptophan; tyrosine.

5) SUN, serum urea nitrogen was estimated as 46.7% of the total serum urea.

**Table 6:** Abundance of hepatic protein associated with ureagenesis (CPS-1) and expression of *mRNA* markers associated with beta oxidation of non-esterified fatty acids (CPT-1 and PGC-1α) in skeletal muscle according to frequency of supplementation during prepartum of grazing primiparous beef cows

Item (arbitrarity units)	Treatment			SEM	p-value1)	
	
	Control	Daily	Infrequent		C vs S	D vs I
CPS-1	0.86	0.81	0.81	0.13	0.81	0.99
CPT-1	8.88	6.97	8.67	0.63	0.27	0.16
PGC-1α	7.03	5.36	6.71	0.90	0.43	0.36

SEM, standard error of the mean; CPS-1, carbamoyl phosphate synthetase I; CPT-1, carnitine palmitoyltransferase 1; PGC-1α, peroxisome proliferator-activated receptor γ coactivator 1α.

1) C vs S = contrast between cows supplemented daily + cows infrequently supplemented versus non supplemented cows; D vs I = contrast between cows supplemented daily versus cows supplemented every three αdays. Values differ significantly at p<0.10.
