# Supplementary material for: Effects of energy-protein supplementation frequency on performance of primiparous grazing beef cows during pre and postpartum
Source: Asian-Australas J Anim Sci. 2020 Feb 25;33(9):1430–43. doi: 10.5713/ajas.19.0784 (PMC7468163; doi:10.5713/ajas.19.0784)
Supplement: Supplementary file 1 [file ajas-19-0784-suppl.pdf]

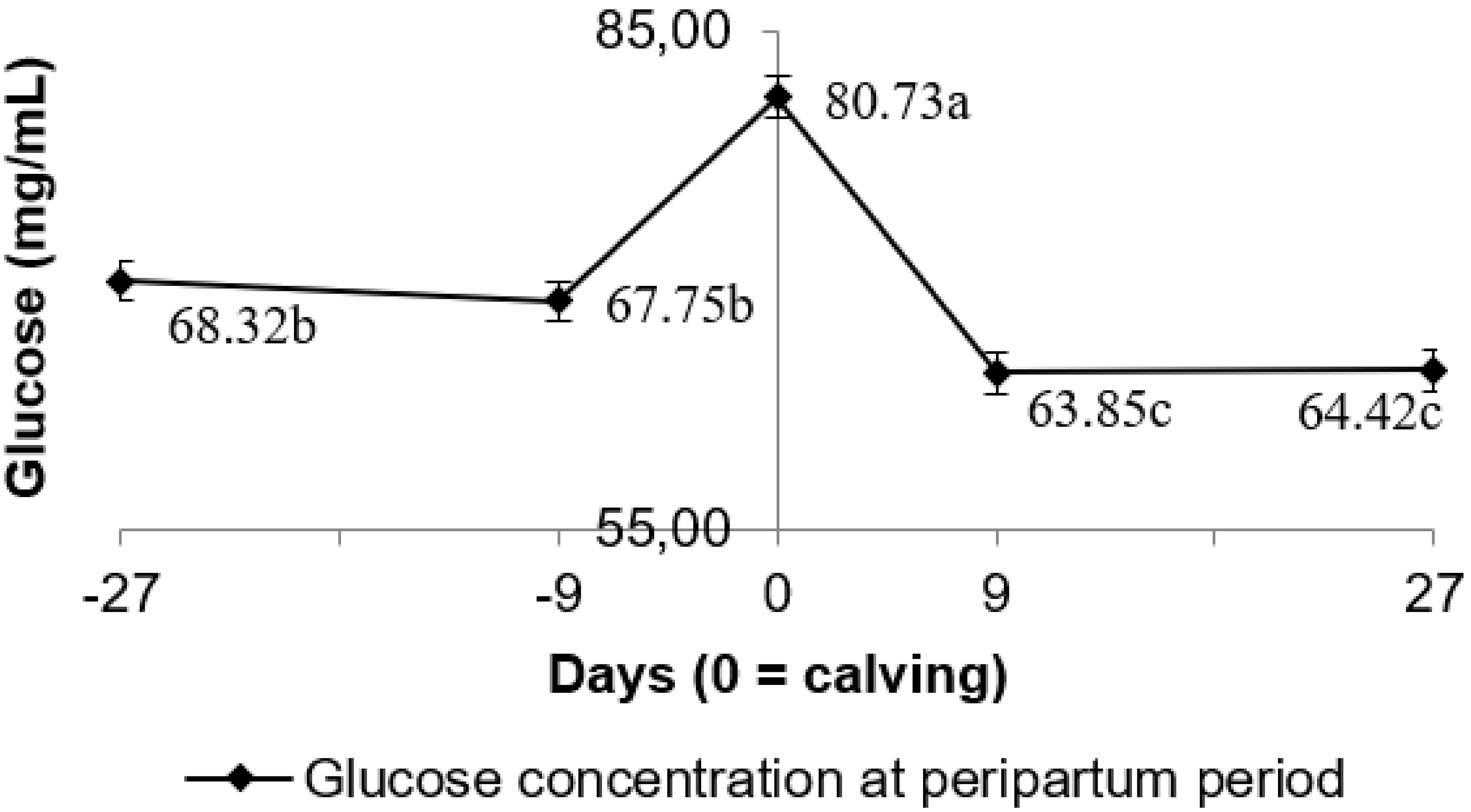

Figure S1. Pattern of glucose concentration during pre (-27 d and -9 d prior to parturition) and postpartum (9 d and 27 d after parturition) of grazing primiparous beef cows. Least square means followed by different letters are different ($p < 0.10$). Blood glucose concentration varies according with sampling time, demonstrating greater concentration at the calving day.

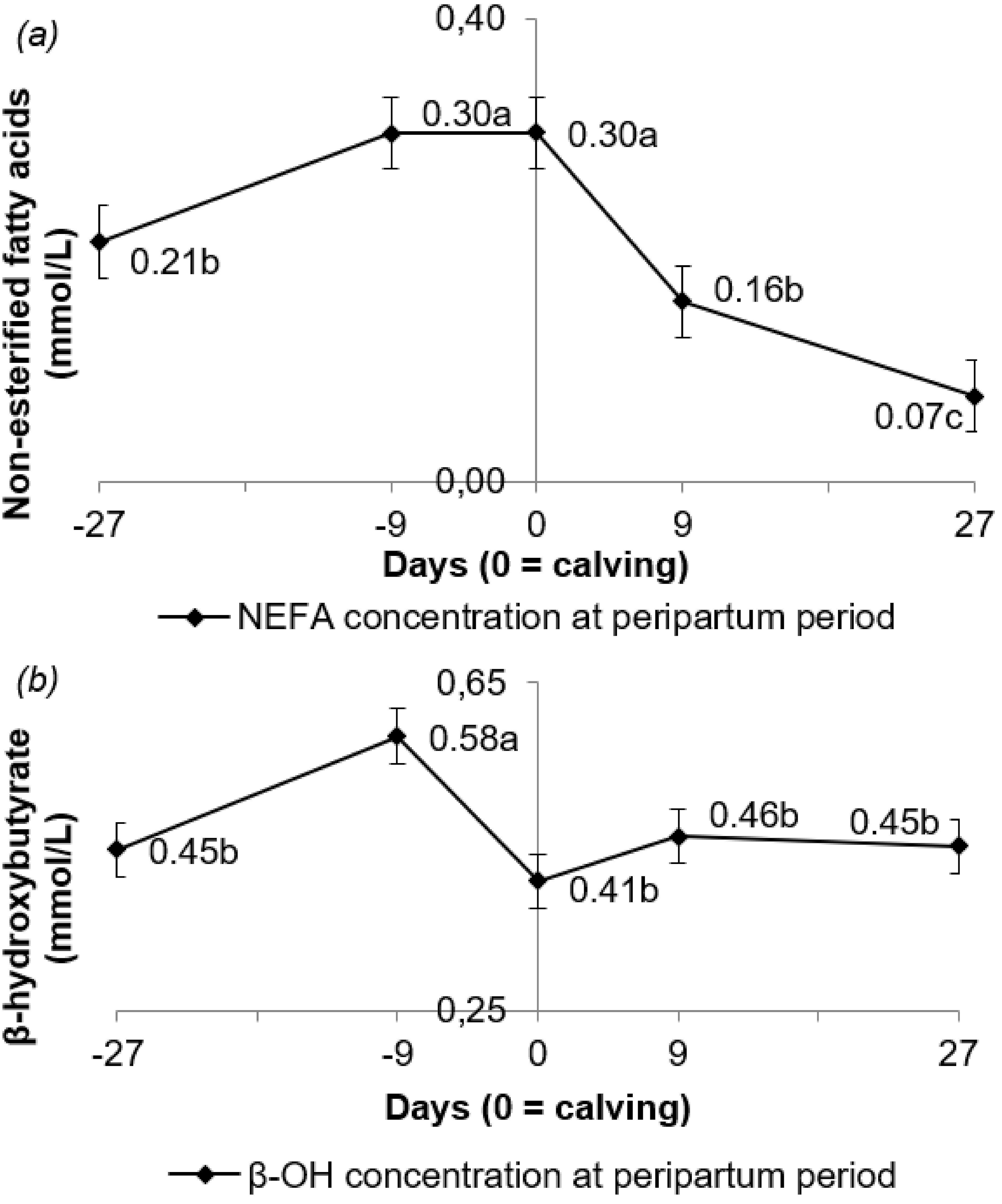

Figure S2. Pattern of (a) non-esterified fatty acids concentration and (b) β-hydroxybutyrate during pre (-27 d and -9 d prior to parturition) and postpartum (9 d and 27 d after parturition) of grazing primiparous beef cows. Least square means followed by different letters are different ($p < 0.10$). Sampling time effect was observed in non-esterified fatty acids (NEFA) and β-hydroxybutyrate (β-OHB) serum levels.

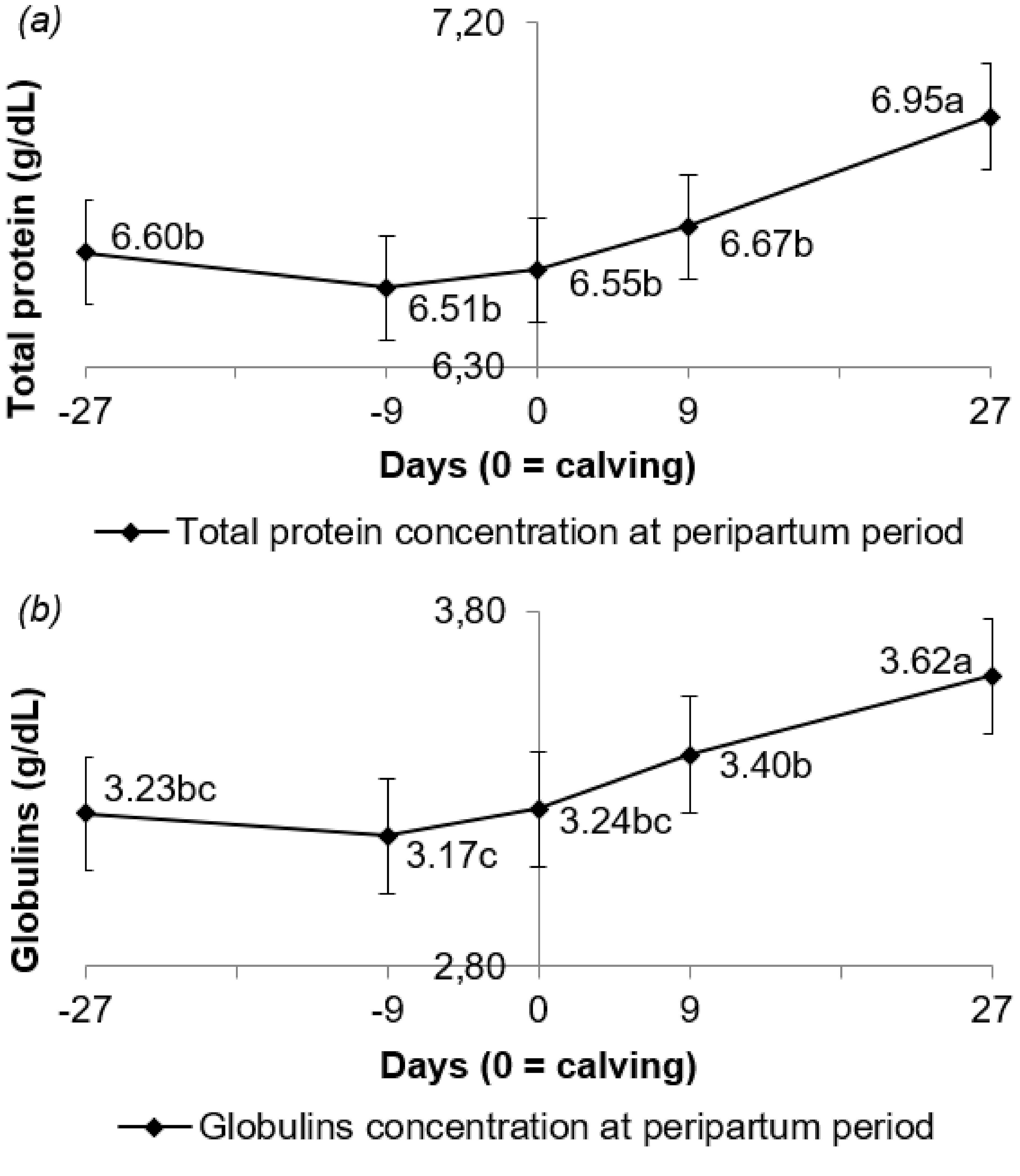

Figure S3. Pattern of (a) total protein and (b) globulins concentration during pre (-27 d and -9 d prior to parturition) and postpartum (9 d and 27 d after parturition) of grazing primiparous beef cows. Least square means followed by different letters are different ($p < 0.10$). Sampling time effect was observed in total protein and globulins levels, demonstrating an increase over time.

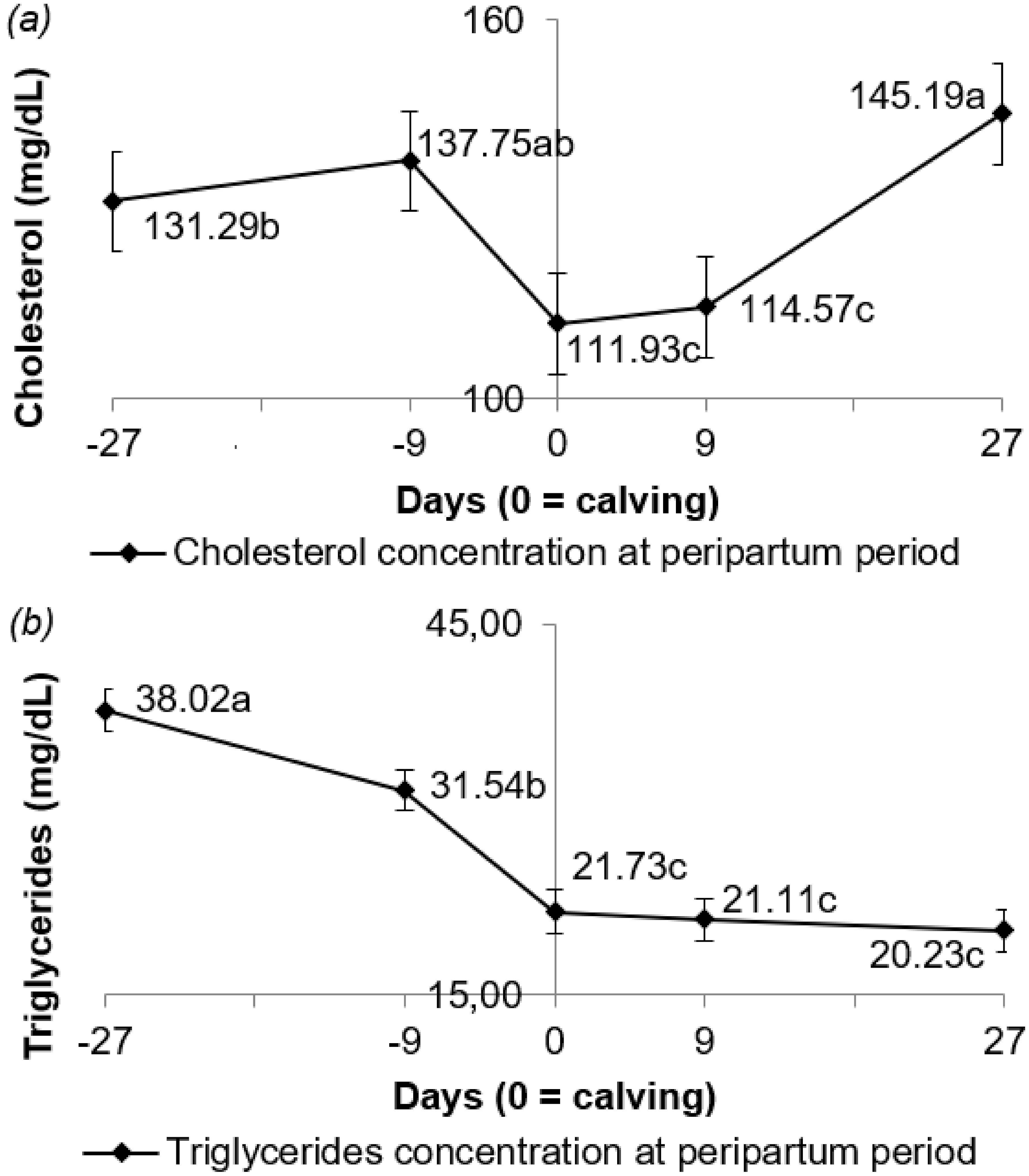

Figure S4. Pattern of (a) cholesterol and (b) triglycerides concentration during pre (-27 d and -9 d prior to parturition) and postpartum (9 d and 27 d after parturition) of grazing primiparous beef cows. Least square means followed by different letters are different ($p < 0.10$). Sampling time effect was observed in cholesterol and triglycerides levels, with an increase or decrease on the concentration over time, respectively.
